# The effect of dietary approaches to stop hypertension and ketogenic diets intervention on serum uric acid concentration: a systematic review and meta-analysis of randomized controlled trials

**DOI:** 10.1038/s41598-023-37672-2

**Published:** 2023-06-28

**Authors:** Sepehr Gohari, Saeed Ghobadi, Alireza Jafari, Hassan Ahangar, Sheida Gohari, Mahsa Mahjani

**Affiliations:** 1 Student Research Center, School of Medicine, Zanjan University of Medical Sciences, Zanjan, Iran; 2 Department of Family Medicine, School of Medicine, Alborz University of Medical Sciences, Alborz, Iran; 3 Institute for Physical Activity and Nutrition (IPAN), School of Exercise and Nutrition Sciences, Deakin University, Melbourne, Australia; 4 Physiology Research Center, Institute of Neuropharmacology, Kerman University of Medical Sciences, Kerman, Iran; 5 Department of Cardiology, School of Medicine, Mousavi Hospital, Zanjan University of Medical Sciences, Zanjan, Iran; 6 Department of Systems Science and Industrial Engineering, State University of New York at Binghamton, Binghamton, NY USA; 7 Endocrine Research Center, School of Medicine, Shahid Beheshti University of Medical Sciences, Tehran, Iran; 8 Student Research Center, School of Medicine, Shahid Beheshti University of Medical Sciences, Tehran, Iran

**Keywords:** Nutrition, Metabolism

## Abstract

Hyperuricemia as a risk factor for metabolic diseases is proved to be profoundly modified by dietary approaches. This systematic review and meta-analysis of randomized control trials (RCT) was conducted to investigate the effect of two nutritional interventions; dietary approaches to stop hypertension (DASH) diet and ketogenic diet (KD) on serum uric acid (UA) concentrations. Our systematic search was for RCTs in which KD or DASH diet were assigned to adults for at least 2 weeks or more. Until March 2023 in Embase, Web of Science, PubMed, and Scopus databases, 10 eligible RCTs that intervened with DASH diet (n = 4) or KD (n = 6) and had provided laboratory data on serum UA were found. Summary effect was calculated by random-effects model. Results from the meta-analysis of the 4 DASH diet RCTs with a total of 590 participants revealed significant decrease in serum UA after at least 4 weeks of interventions (mean difference (MD) = ‒0.25; 95% CI ‒0.4 to ‒0.1 mg/dL; *p* < 0.01; I^2^ = 0%). The pooled meta-analysis of the 6 included RCTs of KD reporting data of 267 participants showed no significant changes in serum UA (MD = 0.26; 95% CI ‒0.47 to 0.98 mg/dL, I^2^ = 95.32%). However, a non-significant reduction of UA in the subgroup analysis of very low-calorie KD (VLCKD) studies (MD = ‒0.04; 95% CI ‒0.29 to 0.22, I^2^ = 0%) was obtained. DASH diet has an ameliorating effect on serum UA and may be recommended for hyperuricemia states such as gout. In addition, we have shown that serum UA level following KD remained unchanged. Although, in view of the heterogeneity across the studies, further investigations are needed to determine the effect of KD and VLKD on serum UA concentrations.

## Introduction

Hyperuricemia is an important risk factor in the development of gout and has strong associations with cardiovascular and metabolic diseases^1^. As uric acid (UA) concentrations are highly dependent to eating habits, weight loss strategies are considered major contributors to decrease the incidence of hyperuricemia^2,3^. Therefore, dietary approaches have been shown to profoundly modify hyperuricemia and improve gout symptoms. Traditionally, purine-rich intake restrictions have been the only cornerstone of dietary strategies^4^.

The interventions of the Dietary Approaches to Stop Hypertension (DASH) diet have emerged to reduce blood pressure and risk of several other cardiometabolic disorders^5,6^, more recently, studies have shown the association of DASH diet with lower UA^7^. DASH diet is comprised of fruit, vegetables, low-fat dairy products and moderate proteins^8^. Furthermore, based on the current observations, it is suggested that high serum UA plays an essential role in the development of hypertension^9^. According to a cross-sectional study each 1 mg/dL increase in serum UA increases the risk of hypertension by 20% in a general population^10^. Although some studies have failed to identify an explicit association^11^.

Ketogenic diet (KD) is of low carbohydrate, high fat proportion and moderate protein which is used widely for the management of neurological disorders and weight loss^12^. KD drives body to burn fat as a substitute of carbohydrate for primary source of energy. Many observational studies have identified a transient hyperuricemia occurs during the ketosis phase in KD^13,14^. The association between the KD and UA concentration has been poorly established. Moreover, serum UA changes with very low carbohydrate ketogenic diets (VLCKD) have been a matter of dispute^15,16^.

In view of the current discrepancies of the effect of KD and DASH diet on serum UA, we have conducted this systematic review and meta-analysis of randomized control trials to determine the overall changes in UA in each dietary approach.

## Materials and methods

The study was performed in accordance with PRISMA statement 2020 for the Preferred Reporting Items for Systematic Reviews and Meta Analyses^17^ (Supplementary file, Section A). The protocol was already registered in PROSPERO, https://www.crd.york.ac.uk/PROSPERO, ID: [CRD42022355814].

### Inclusion criteria

Two different authors (SEP.G and M.M) carefully screened all searches according to the titles and abstracts to find eligible studies after removal of all duplicate reports. Next, all eligible studies were categorized based on their findings and contents. Authors (SEP.G and SA.G) independently checked all screening articles. The studies that met the following criteria were included in our meta-analysis: (1) Randomized controlled trial (RCT) studies, (2) populations aged 18 years old and higher (3) compared DASH or ketogenic diet with a control group, (4) Provide serum UA for each group.

The exclusion criteria included: (1) Dietary interventions on pregnant participants (2) participants with specific diseases that potentially carries alterations in serum UA (e.g., Malignancy and epilepsy), (3) the intervention period was less than 2 weeks (4) the state of ketosis was not achieved (for KD trials).

### Literature search strategy

The authors systematically searched online databases including Embase, Web of Science, PubMed, and Scopus for relevant articles until March 2023. The searches were conducted using the following main Medline keywords: (((Dietary Approaches To Stop Hypertension[Title/Abstract]) OR (Dietary Approaches To Stop Hypertension[mh])) OR (((Diet, Ketogenic[mh]) OR (Diet, Ketogenic[Title/Abstract])))) AND (((((((((((((((((randomized controlled trial[pt]) OR (controlled clinical trial[pt])) OR (randomized controlled trial[mh])) OR (random allocation[mh])) OR (double blind method[mh])) OR (single blind method[mh])) OR (clinical trial[pt])) OR ((clin*[Title/Abstract]) (trial*[Title/Abstract]))) OR (((singl*[Title/Abstract] OR doubl*[Title/Abstract] OR trebl*[Title/Abstract] OR tripl*[Title/Abstract]) (blind*[Title/Abstract] OR mask*[Title/Abstract])))) OR (placebos[mh])) OR (placebo*[Title/Abstract])) OR (random*[Title/Abstract])) OR (research design[mh])) OR (comparative study[pt])) OR (follow up studies[mh])) OR (prospective studies[mh])) OR ((control*[Title/Abstract] OR prospectiv*[Title/Abstract] OR volunteer*[Title/Abstract]))) with all their sub-trees in different combinations. There were no restrictions on language. Additionally, a manual search of references of related papers was performed to include any possible missed articles. The detailed information regarding the search strategy is presented in supplementary file (Section B).

### Data extraction

Two authors (SEP.G, M.M) were responsible for data extraction, using a prepared checklist consists of the following data: name of the first author, country of origin, year of publication, study design, study duration, study population, demographics and past medical history of study population, type of the intervention, serum UA at baseline, study period and intergroup changes, and the related data for further steps were extracted.

### Risk of bias and certainty of evidence assessment

The quality of evidence in each article was methodologically assessed by two independent authors (SA.G and M.M) using the Cochrane risk-of-bias tool for randomized trials (RoB-2)^18^. The domains of this scale are listed as randomization process, deviation from intended intervention, missing outcome data, measurement of the outcome, and the selection of the reported result. Based on the guideline, studies are categorized into low, high, and some concerns for the risk of bias. Disagreements between authors were resolved through discussion with two other authors (A.J, SH.G) who were blinded to the previous assessments. GRADEpro GDT software^19^ was used to rate the certainty of evidence in the meta-analysis for each diets individually. The GRADE (Grading of Recommendations Assessment, Development, and Evaluation) system assess the quality of evidence based on areas of study design, risk of bias, inconsistency, indirectness and imprecision and categorizes the corresponding results into: high, moderate, low, and very low^20^.

### Statistical analysis

We used paired-wised meta-analyses to obtain mean difference (MD) and 95% confidence interval (CI) via random-effects modelling with restricted maximum likelihood. Whenever SD _change_ was not reported, we used [(SD_baseline_^2^ + SD_final_^2^)–(2 × R × SD_baseline_ × SD_final_)]^21^. We also used Hartung-Knapp adjustment to provide more conservative analysis^22^. Statistical heterogeneity between studies was investigated using Higgins I^2^ statistics (low 25%, moderate 50%, and high > 50%), τ^2^, and the Cochrane Q test (*P* < 0.1)^23^. We conduct subgroup analyses based on different amounts of calory intake in KD to investigate the source of heterogeneity. The potential for publication bias was not assessed (less than 10 study included)^24^. We conducted a sensitivity analysis by excluding or including studies one by one to evaluate the impact of individual studies on the overall estimate. Cohenʼs Kappa statistic was used to assess the inter-reviewer’s agreement in finding the studies, data extraction, and quality assessment^25^. A statistical significance was considered Two-tailed *P*-value < 0.05. All analyses were fulfilled by using Stata (Stata Statistical Software: Release V.14. College Station, Texas, USA: StataCorp LLC)^26^.

## Results

### Study selection

**Figure 1:**
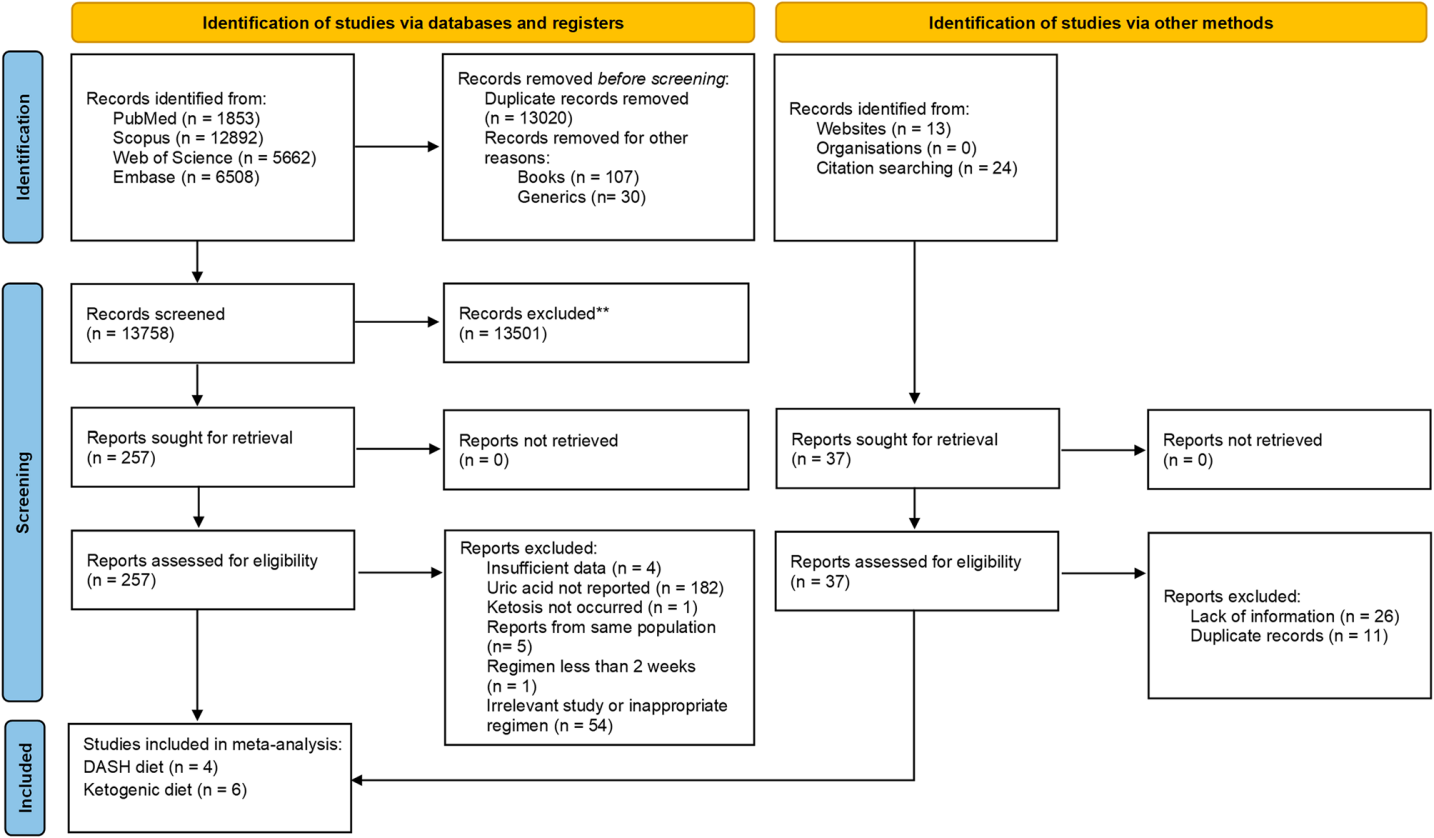
PRISMA flow-diagram. * The excluded articles comprised of reviews, irrelevant title or topic, letters, conference abstracts, case reports, non-randomized studies, studies on pregnant or pediatric subjects, studies in which the recruited patients had underling disease which potentially interact with serum uric acid concentration (e.g. malignancy or epilepsy), animal model or *in-vitro* studies. DASH: Dietary approaches to stop hypertension.

**Figure 2:**
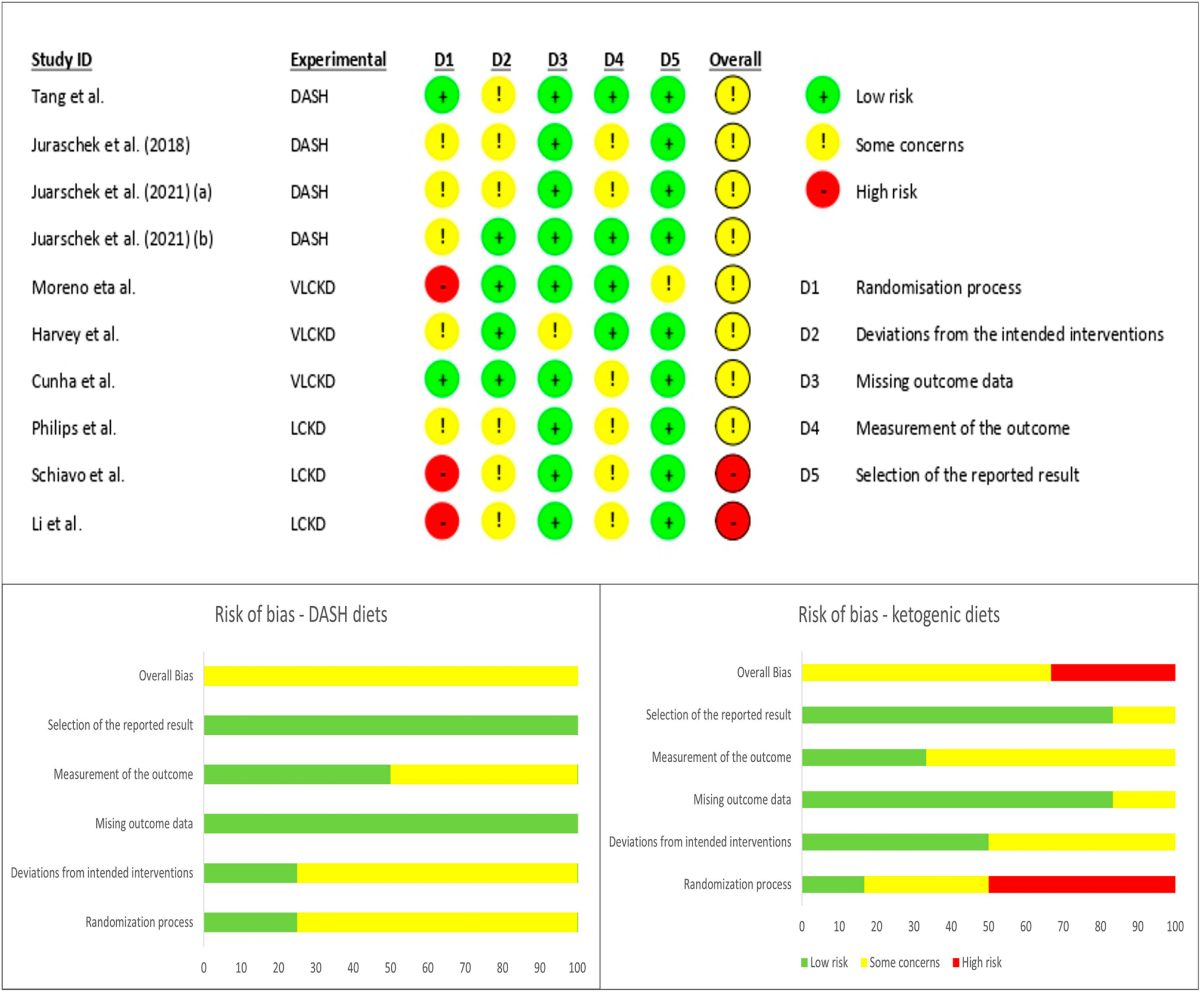
Manuscript quality assessment using ROB-2 tool.

A total of 26,915 studies were obtained with the designed search strategy. After excluding duplicates and irrelevant publications, 257 studies were found to be eligible for full text assessment. No foreign language manuscript was chosen for full text assessment. Finally, 10 studies (4 RCTs for DASH and 6 for ketogenic diets) were included in the meta-analysis. The detailed information regarding the excluded studied is presented in Fig. 1. Based on the quality assessment results, the overall risk of bias for DASH diet and KD studies were considered intermediate and high, respectively. In 9 studies the randomization was not concealed (Fig. 2). The GRADE assessment revealed that the quality of evidence in the meta-analysis of DASH diets was high while the evidence for KD diets was found to be very low (Supplementary file, Section C). The agreement proportion among authors in data extraction and selection and quality assessment based on kappa coefficients was 85% that implies a fair agreement between researchers.

### Study characteristics and nutritional interventions

#### DASH

Through our systematic search a total of 4 relevant RCTs with the DASH diet were identified as eligible to be included in the meta-analysis^7,27–29^. The characteristics of studies and patients are summarized in Table 1. A total of 590 participants had been randomized into DASH diet and control arms. Among the participants that were assigned to DASH diet arms, an approximate 40% had a baseline hyperuricemia and 45% of them were hypertensive. Study sample sizes ranged from 43 to 327 and the duration of each DASH diet varied from 4 to 12 weeks. DASH diets in all 4 studies consisted of fruits, vegetables, and low-fat dairy foods and reduced consumption of saturated fat, total fat, and cholesterol along with whole grains, poultry, fish, and nuts and restricted red meat, sweets, and sugar-containing beverages. The included studies were dietician-directed, and participants had been closely monitored for higher adherence to the assigned diet. Randomization was computer-generated in one study^27^. The comparator groups received different types of weight maintenance diets. Two of the control arms in DASH studies were typical American diet and had the components of the average intake level in the US^7,27^ and the other two were the individuals’ routine diet^28,29^. Studies had provided allowances of 30 to 105$ per week for grocery shopping.

**Table 1:** Study and baseline characteristics of DASH diets.

Author/year	Design/country	Exclusion of the urate-lowering drugs	Duration (weeks)	Total sample size/population	Baseline characteristics of interventional group				Dietary facts		Changes in UA; DASH vs control^6^ (mg/dL, 95% CI)
					Gender, female (%)	Age, Mean (y)	HTN (%)	High baseline UA (%)	Intervention	Control	
Tang et al.^27^	RCT/USA	N/A	12	103/Adults with pre- or stage 1 HTN	62.7	49.9	29.4	6.6^1^	DASH^5^ diet	Typical isocaloric American diet	− 0.40 (− 0.79, − 0.01)
Juraschek et al.^28^	RCT/USA	No	8	117/Adult African-Americans with controlled HTN	65	59.2	100	30^2^	DASH-plus diet: coach-directed, 30$/week allowance limited to high K foods (fruits, vegetables, nuts, or dried beans)	A DASH brochure & 30$/week allowance	− 0.01 (− 0.39, 0.38)
Juraschek et al.^29^	RCT/USA	Yes	4	43/Adults with gout (self-reported) & a UA concentration > 7 mg/dL	18	42.5	50	100^3^	DASH diet with 105$/week allowance of dietitian-directed groceries	self-directed grocery shopping with 15$/day allowance	− 0.51 (− 1.16, 0.13)
Juraschek et al.^7^	RCT/USA	No	8	327/Adults with pre- or stage 1 HTN	52.7	44.8	24.5	23.0^4^	DASH diet with K, Mg & Ca at the 75th percentile levels of U.S. consumption, and high in fiber and protein	K, Mg, and Ca levels matching the 25th percentile of U.S. consumption, & macronutrient and fiber at the average intake levels in the U.S	− 0.25 (− 0.43, − 0.08)

RCT: Randomized Control Trial, HTN: Hypertension, UA: Uric Acid, DASH: Dietary Approaches to Stop Hypertension, CI: Confidence Interval, K: potassium, Mg: magnesium, Ca: calcium.

^1^UA ≥ 6 mg/dl.

^2^UA > 7 mg/dl.

^3^UA ≥ 7 mg/dl.

^4^UA > 6 mg/dl for women or > 7 mg/dl for men.

^5^All mentioned DASH diets consist of fruits, vegetables, and low-fat dairy foods and reduced consumption of saturated fat, total fat, and cholesterol along with whole grains, poultry, fish, and nuts and restricted red meat, sweets, and sugar-containing beverages.

^6^As mentioned in the articles.

#### KD

Table 2 is the summarized characteristics of included studies for KD. The six included trials were comprised of 267 participants that 119 of which (three RCTs) were assigned to a VLCKD or control diet^15,16,30^, while, the remaining individuals (148 of the other three RCTs)^31–33^ were intervened with either LCKD or a comparator diet. All the participants were adults and aged from 18 to 65 years and their follow-up period varied from 8 to 16 weeks. A VLCKD encompasses daily calorie intake of about 600–800 kcal and is low in carbohydrates (< 50 g daily from vegetables) and lipids (only 10 g of olive oil per day). Proteins were limited to 0.8 and 1.2 g per each kg of ideal body weight as to ensure meeting the minimal body requirements and to prevent the loss of lean mass. The compositions of LCKD were high daily fat (135–150 g), restricted carbohydrates (∼4% or 16–50 g/day), and 1200–1750 kcal/day.

**Table 2:** Study and baseline characteristic of ketogenic diets.

Author/year	Design	country	Duration(weeks)	Total sample size	population	Baseline characteristics of interventional group			Dietary facts		Changes in UA; KD vs control^1^ (mg/dL, 95% CI)
						Gender, female (%)	Age, range (y)	BMI, Mean (kg/m^2^)	Ketogenic diet type	Control	
Moreno et al.^30^	RCT	Spain	8	53	Adult participants with obesity (BMI ≥ 30 kg/m^2^)	81.4	18–65	35.1	**VLCKD**; (600–800 kcal/day), low carbohydrates (50 g daily from vegetables) and lipids (only 10 g of olive oil /day), proteins (0.8 and 1.2 g per each Kg of ideal body weight)	**LCD**; 1,400 and 1,800 kcal/day, carbohydrates: 45–55%, Proteins: 15–25%, and fat: 25–35%	-0.13 (-0.49, 0.23)
Harvey et al.^15^	RCT	New Zealand	12	27	Healthy individuals	64.3	25–49	25.5	**VLCKD;** Carbohydrate: 5%, protein limited to 1.4 g/kg/day	**LCD**; Carbohydrate: 15%, protein limited to 1.4 g/kg/day	0.15 (-0.47, 0.77)
Phillipset al.^31^	RCT	New Zealand	8	47	patients with Parkinson’s disease	29	40–50	27.8	**LCKD**; 1,750 kcal/day composed of 152 g of fat (67 g saturated), 75 g of protein, 16 g of carbohydrate, and 11 g of fiber	**Low fat diet**; 1,750 kcal/day composed of 42 g of fat (10 g saturated), 75 g of protein, 246 g net carbohydrate, and 33 g of fiber	0.84 (0.45, 1.23)
Cunha et al.^16^	RCT	Brazil	8	39	Adults with obesity (BMI ≥ 30 kg/m^2^)	N/A	N/A	37.1	**VLCKD**; 600–800 kcal/day, low in carbohydrates (< 50 g daily from vegetables) and lipids (only 10 g of olive oil per day), proteins: 0.8 to 1.2 g/kg of ideal body weight	**LCD**; 15% below the total metabolic expenditure of each individual, carbohydrates: 45–55%, proteins: 15–25%, and fat: 25–35%, 20–40 g/day of fiber	0.01 (-0.44, 0.46)
Schiavo et al.^32^	RCT	Italy	16	48	Obese individual undergoing The Elipse™ intragastric balloon (BMI ≥ 27 kg/m^2^)	50	18–65	37.8	**LCKD**; 1200 kcal/day, fat: 71%, carbohydrates: 4%, protein: 25%	**LCD**; 1200 kcal/day, fat: 17%, carbohydrates: 40%, protein: 43%	-1.2 (-2.12, -0.28)
Li et al.^33^	RCT	China	12	53	Overweight and obese patients with BMI ≥ 25 kg/m^2^, newly diagnosed as T2DM	N/A	18–50	29.0	**LCKD**; 1500 ± 50 kcal/day, carbohydrate: 30–50 g, protein: 60 g, fat: 130 g	**Diabetic diet**; 1500 ± 50 kcal/day, carbohydrate: 250–280 g, protein: 60 g, fat: 20 g	1.55 (1.39, 1.71)

RCT: Randomized Control Trial, BMI: Body Mass Index, VLCKD: Very-Low Calorie Ketogenic Diet, LCD: Low-Carbohydrate Diet, UA: Uric Acid, KD: Ketogenic Diet CI: Confidence Interval, LCKD; Low-Carbohydrate Ketogenic Diet.

^1^As mentioned in the articles.

#### Meta-analysis of DASH and UA

**Figure 3:**
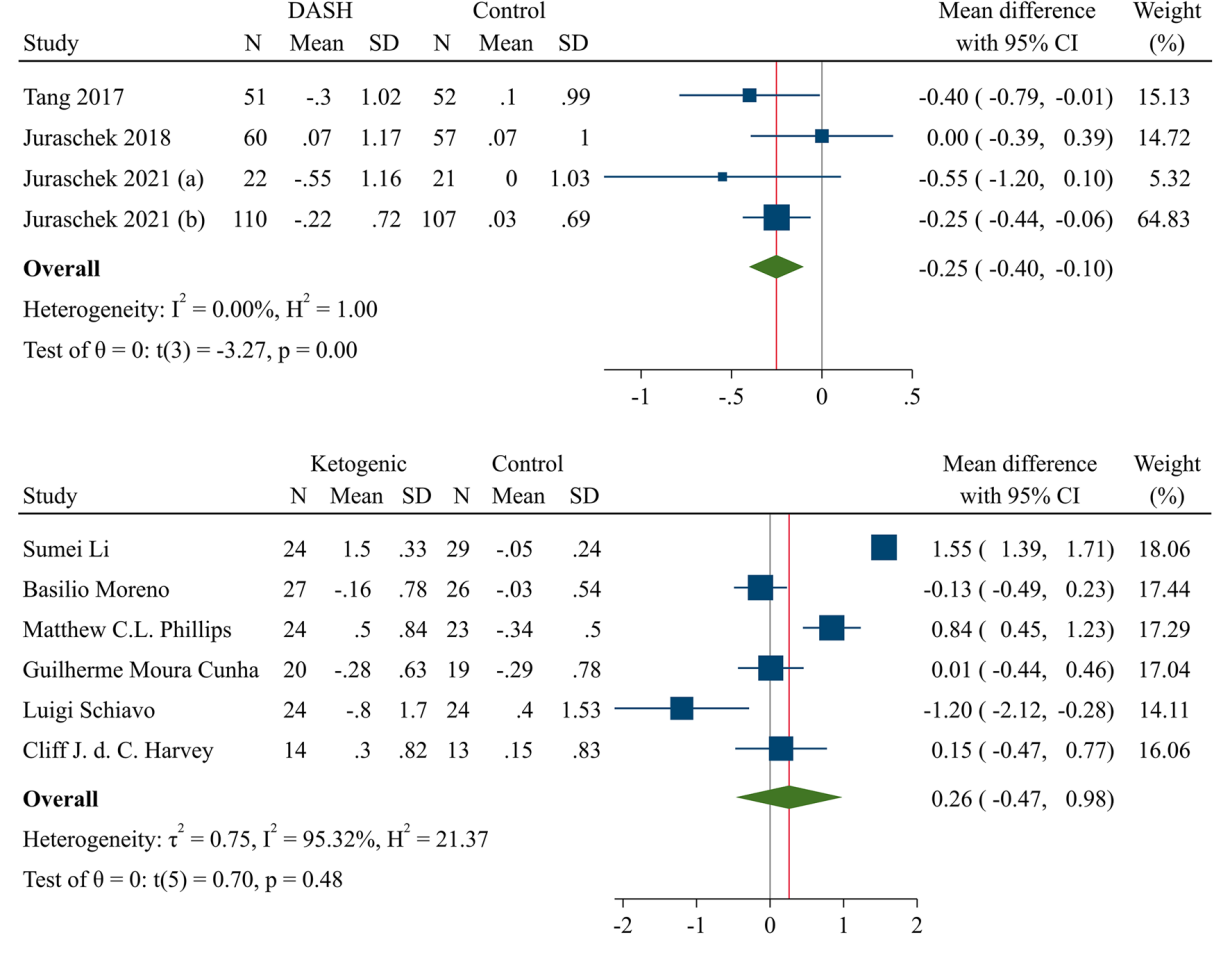
Forest plot of the meta-analysis of 4 DASH diet and 6 KD RCTs and changes in serum UA. DASH: Dietary approaches to stop hypertension; KD: Ketogenic diet; UA: Uric acid; RCT: Randomized controlled trial; CI: Confidence interval.

The pooled analysis revealed a significant reduction in serum UA following the DASH diet (*P* < 0.01). The pooled mean difference (MD) of UA from baseline was −0.25 (95% CI −0.40 to −0.10; H–K: −0.50 to −0.01) with a very low heterogeneity among the studies (I^2^ = 0%, 95%CI 0–85%; H^2^ = 1.00). The forest plot of DASH diet and changes in UA in addition to leave-one out sensitivity analysis is depicted in Fig. 3 and supplementary file (Section D, Fig. 1), respectively.

#### Meta-analysis of KD and UA

**Figure 4:**
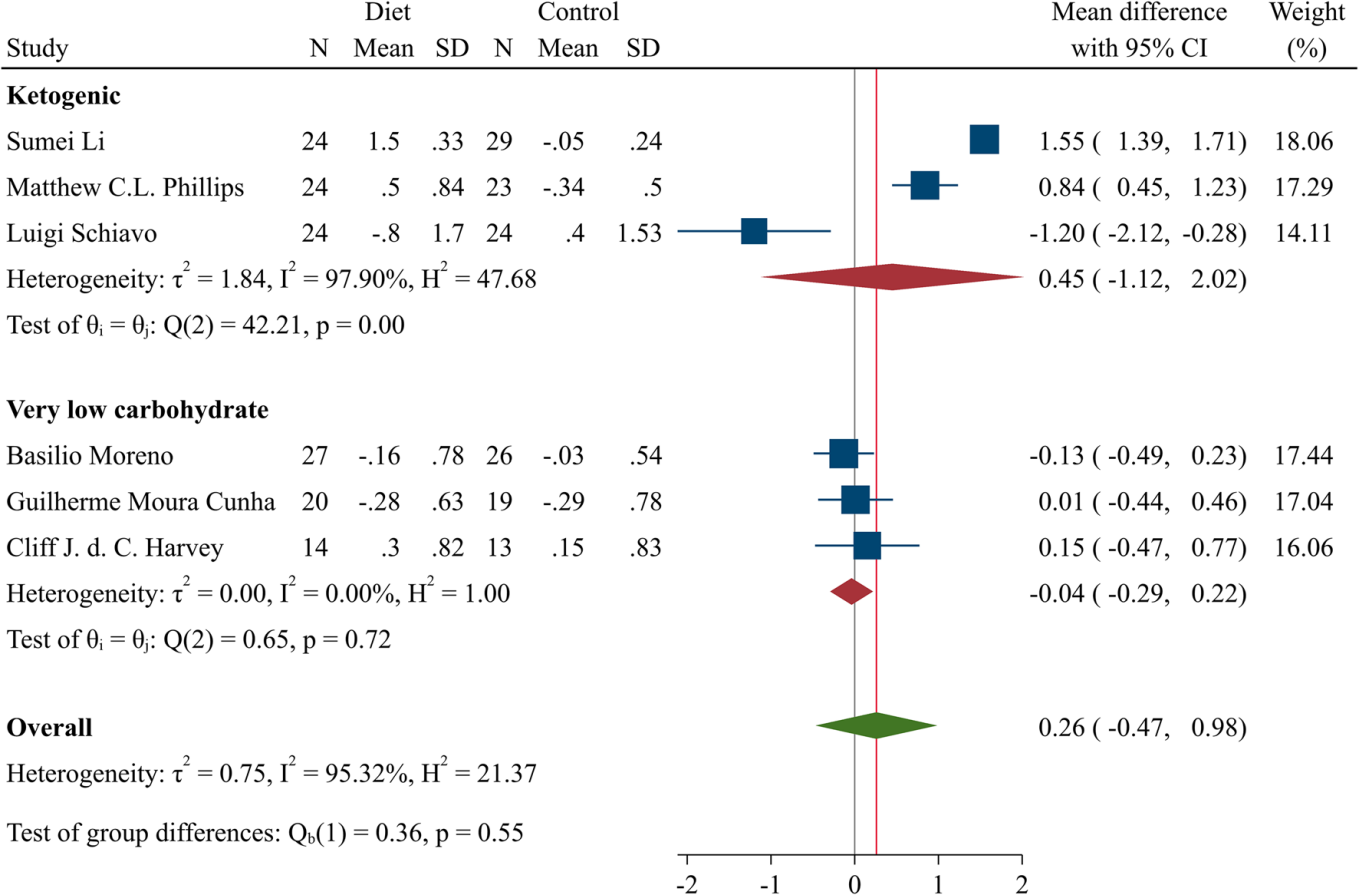
Forest plot of two KD subgroups; LCKD and VLCKD. KD: Ketogenic diet; VLCKD: Very low-calory ketogenic diet; CI: Confidence interval.

The overall meta-analysis of KD studies is demonstrated in Fig. 3. KD intervention showed a neutral effect on serum UA (MD = 0.26; 95% CI: −0.47–0.98, H–K: −0.70–1.22; I^2^ = 95.32%, 95% CI 94–98%). Results of the subgroup analysis of VLCKD studies showed a non-significant reduction in serum UA (MD = −0.04; 95% CI −0.29–0.22, H–K: −0.60–0.52; I^2^ = 0%, 95% CI 0–90%) (Fig. 4). The leave-one out sensitivity analysis of the KD is presented in supplementary file (Section D, Fig. 2).

## Discussion

This present meta-analysis Discussed the effects of the two most popular diets, The DASH diet and KD, on the serum UA level. Among all the dietary plans, we were obliged to choose these two diets as limited RCTs were available in databases on the effect of other dietary strategies such as vegetarian and Mediterranean on serum UA. The meta-analysis of DASH diet trials showed that despite their different control groups, overall, DASH diet interventions significantly reduced serum UA. Although, non-significant changes in KD on UA levels were obtained.

### DASH diet and UA

The meta-analysis of DASH diet demonstrated a significant reduction in serum UA with a low level of heterogeneity. That is probably because all the four included RCTs were conducted in the US. Plus, the assigned diets in both intervention and control arms had great similarities. Although one study^29^ included individuals with gout, the rest of them did not have any active use or plan for urate-lowering medications. Besides, all the studies excluded participants with any underlying disease or use of any medications that could potentially affect serum UA. By extension, the small differences between study populations may have not affected the main results.

DASH diet encompasses nutrients that are known to be inversely associated with UA concentration. Foods containing lower glycemic index carbohydrates (e.g., dairy products and some fruits) have reduced UA independent from changes in glucose and insulin levels. As mentioned above, DASH diet contains a high content of low-fat dairy products. Milk-origin proteins, casein and lactalbumin, are purine-free and their role in lowering serum UA has already been examined in several studies. Other components of this diet such as vitamin C have shown similar effects^34^.

Hyperuricemia caused hypertension in almost all clinical studies^9^. The deleterious effects of hyperuricemia on kidneys are defined as the main pathophysiological mechanisms in this regard. The impairment of renal vessels’ endothelium by the pro-inflammatory response elicited by urate crystals as well as the endothelial dysfunction caused by UA through the blockade of nitrite oxide production are proposed pathways in the pathogenesis of hypertension^35,36^. Therefore, UA reduction can improve hypertension management.

The non-significant results of some studies can be explained by several factors^28,29^. The UA reduction rate with DASH diet was much more prominent in a subgroup of participants with hyperuricemia, although in a population with normal or low UA levels, limited change might have been documented; this notion warrants stronger evidence by future studies.

It is important to note that the effect of antihypertensive drugs such as beta-blockers and alpha 1-blockers in urate reduction and the increased urate reabsorption by thiazides in the included studies may have confounded each study’s results^37^. Although, there were limited studies available on the UA change in serum, considering the very low heterogeneity across DASH studies, there is less possibility for any confounding factor that would concern our results, however, they warrant more large-scale RCTs.

### Ketogenic diet and UA

Our meta-analysis of the available literature has shown that KD has failed to alter UA levels. These findings are in accordance with a non-randomized study among type 2 diabetic patients, where mean UA in a KD intervention had a transient rise until day 70 and eventually, remained unchanged after one year^38^. Contrary to the cited results, another KD trial found decreased UA levels on week 24 of intervention^39^. Most of these studies noted a transient hyperuricemia which is primarily present in the early course of the KD initiation; such as the significant serum UA elevation observed in healthy non-obese men 5 days following KD^40^. The hypothesis for the elevated serum UA would be that in KD, when glucose availability drops, other metabolic processes such as ketogenesis rise to compensate for the energy production. Ketone bodies (Beta-hydroxybutyrate and acetoacetate) are products of this increased ketosis^41^. During a ketogenic state, with the accumulation of ketone bodies, the constant gradient of intracellular anions compels urate transporter 1 (URAT1) to reabsorb urate into the cells^42^. Prior studies have shown that the activity of a Na^+^-monocarboxylic acid anions co-transporter which recycles theses anions from apical membrane of tubular lumen into peritubular cells can be inhibited by short chain fatty acids (Butyrate, propionate, acetate)^43,44^. When fatty acids are metabolized in the liver in prolonged low-calorie intake in KD, their inhibitory effect diminishes, however, in most cases UA levels return to baseline values. Although this transient characteristic of ketogenic-induced hyperuricemia has not been clarified, we postulate that with regard to the paradoxical effect of uricosuric drugs in low doses, the regulatory effect of ketone bodies on URAT1 might exhibit a similar pattern^45^.

On the other hand, lower insulin and glycemic index have uricosuric effects and significantly decrease the expression of URAT1^46,47^. The simultaneous impact of low insulin release, consuming low glycemic index foods and increased ketone bodies might contribute to the observed transient pattern of hyperuricemia in KD.

Considering the direct association between purine-rich foods consumption and hyperuricemia, the protein intake between experimental arms in all the six studies included in KD meta-analysis was within a narrow range. The results of VLCKD studies, although limited, were found to relatively favor the reduction of serum UA with a lower heterogeneity compared to the subgroup meta-analysis of low carbohydrate KD (LCKD) studies. The differences between heterogeneities may be attributed to the greater similarities between control groups in VLCKD studies. Moreover, the calorie intake, protein, carbohydrate, and fat components in intervention groups of VLCKD subgroup meta-analysis were more consistent than in LCKD trials. The decreased level of UA during VLCKD may be explained through the body’s struggle to maintain the circulatory ketone bodies in the settings of starvation or near-fasting diets by decreasing their clearance in the kidney^48,49^. Due to the increased intracellular anions, inevitably, urate, which is also an anion, is excreted through organic anion transporter into the urine^50,51^. More well-controlled studies are needed to enroll gout and hyperuricemic patients to evaluate the urate-lowering aspect of KD. Such fluctuations of UA within the ketosis phase raise concern as to whether KD increases the risk of an acute attack of gouty flares. Likewise, the long-term impact of KD on the urate profile is still a topic to be determined.

Furthermore, as there were few papers on KD and alterations in UA, in addition to the remarkable heterogeneity between studies, whether KD can change serum UA concentrations is still debatable and demands more studies. Even though uricosuric drugs are the mainstay of treatments, proper nutritional recommendations, alongside therapeutic strategies, can attenuate the pathophysiological consequences of hyperuricemia.

To the best of our knowledge, this is the first meta-analysis on the effect of DASH diet and KD on serum UA. Another strength of this report is the inclusion of RCT studies. Still, we acknowledge some limitations. First, although all studies declared to be RCTs, in most of them the randomization process was not thoroughly stated, and the blinding was obscure. In addition, the sample sizes were relatively small. Apart from the inherent limitations in individual studies, the main limitation of the DASH meta-analysis was that despite very low heterogeneity between studies, the population in one study comprised of patients with gout. All the included studies in DASH meta-analysis assigned the standard dietary regimen to the interventional arms, while the detailed nutrition components for the control groups were undefined. We observed that considerable heterogeneity across KD studies may have been attributed to the differences in calorie, carbohydrate, and fat intakes between the studies. In the VLCKD subgroup meta-analysis the calorie intake in intervention groups was quite similar and less heterogeneity was thus obtained. Another major limitation in dietary intervention trials is the uncertainty of the participants’ adherence to the diets, and the assessment of whether or not the pooled population adhered to the prescribed diet was not feasible since few studies reported data on this issue. Finally, we included a few studies for each meta-analysis, which was mainly because of the limited data available in the literature.

## Conclusion

Our results have shown that DASH diet has an attenuating effect on serum UA and can be routinely recommended for patients with hyperuricemia. It’s important to design approaches for more dietary adherence to DASH diet with respect to its several health benefits. Moreover, although serum UA did not change with KD, the meta-analysis of VLCKD studies was suggestive of the UA lowering potential of such dietary intervention which needs future confirmatory research.

Future directions.

- To perform dose–response analysis for the effect of DASH diet on serum UA, future studies are sought.
- More studies with more dietary and population consistency should be designed to have more similar study weights for sensitivity analysis.
- In order to determine the most beneficial diet for serum UA reduction, future meta-analyses on other dietary interventions such as Mediterranean, vegetarian, etc. are needed.

## Supplementary Information

Supplementary Information.

## Data Availability

The data/information supporting this study is available from the corresponding author upon reasonable request.

## Abbreviations

UA: Uric acid
DASH: Dietary approaches to stop hypertension
KD: Ketogenic diet
VLCKD: Very low-calorie ketogenic diet
LCKD: Low carbohydrate ketogenic diet
RCT: Randomized controlled trial
MD: Mean difference
URAT1: Urate transporter 1
